# Different Expressions of HIF-1α and Metabolism in Brain and Major Visceral Organs of Acute Hypoxic Mice

**DOI:** 10.3390/ijms22136705

**Published:** 2021-06-23

**Authors:** Lu Xu, Hua Song, Qi Qiu, Ting Jiang, Pingyun Ge, Zaiji Su, Wenhui Ma, Ran Zhang, Caihua Huang, Shanhua Li, Donghai Lin, Jiaxing Zhang

**Affiliations:** 1 Institute of Brain Diseases and Cognition, Medical College of Xiamen University, Xiamen 361102, China; xlxmu@stu.xmu.edu.cn (L.X.); qiuqi9592@stu.xmu.edu.cn (Q.Q.); mawenhui7@126.com (W.M.); zhangran2008@xmu.edu.cn (R.Z.); 2 Fujian Provincial Key Laboratory of Innovative Drug Target Research, School of Pharmaceutical Sciences, Xiamen University, Xiamen 361102, China; songhua@xmu.edu.cn (H.S.); gxx_1128@163.com (P.G.); 32320191153407@stu.xmu.edu.cn (Z.S.); 3 Key Laboratory for Chemical Biology of Fujian Province, College of Chemistry and Chemical Engineering, Xiamen University, Xiamen 361005, China; jt127721@126.com; 4 Research and Communication Center of Exercise and Health, Xiamen University of Technology, Xiamen 361024, China; huangcaihua@xmut.edu.cn

**Keywords:** hypoxia, metabolism, HIF-1α, brain, liver, kidney

## Abstract

Hypoxia is associated with clinical diseases. Extreme hypoxia leads to multiple organs failure. However, the different effects of hypoxia on brain and visceral organs still need to be clarified, and moreover, characteristics in vulnerable organs suffering from hypoxia remain elusive. In the present study, we first aimed to figure out the hypoxic sensitivity of organs. Adult male mice were exposed to 6% O_2_ or 8% O_2_ for 6 h. Control mice were raised under normoxic conditions. In vivo and in vitro imaging of anti-HIF-1α-NMs-cy5.5 nanocomposites showed that the expression level of hypoxia-inducible factor (HIF-1α) was the highest in the liver, followed by kidney and brain. HIF-1α was detected in the hepatocytes of liver, distal convoluted tubules of kidney and neurons of cerebral cortex. The liver, kidney and brain showed distinct metabolic profiles but an identical change in glutamate. Compared with kidney and brain, the liver had more characteristic metabolites and more disturbed metabolic pathways related to glutaminolysis and glycolysis. The level of O-phosphocholine, GTP, NAD and aspartate were upregulated in hypoxic mice brain, which displayed significant positive correlations with the locomotor activity in control mice, but not in hypoxic mice with impaired locomotor activities. Taken together, the liver, kidney and brain are the three main organs of the body that are strongly respond to acute hypoxia, and the liver exhibited the highest hypoxic sensitivity. The metabolic disorders appear to underlie the physiological function changes.

## 1. Introduction

Oxygen is critical to the body. Acute hypoxia is common in clinical diseases, such as birth asphyxia and traumatic injury that limits respiration, and in daily life, such as pilots and mineworkers with short O_2_ supply and people who travel fast to high altitudes. Systemic hypoxia drives multi-organ damage such as cognitive decline [1], pulmonary hypertension [2], heart failure [3,4], liver injury [5] and renal damage [6]. Each organ has its own oxygen requirement to maintain tissue homeostasis [7,8]. Different organs have varying tolerance to hypoxia, partially due to both the systemic redistribution of the inadequate O_2_ supply in favor of vital organs and the distinctive intrinsic organ responses [9,10]. Expectedly, comparison and understanding of the responses of vital organs to acute hypoxia might improve the treatment of diseases associated with tissue hypoxia.

After acute exposure to hypoxia, the heart rate increases immediately, which elevates cardiac output and maintains systemic oxygen supply, with no cardiomyocyte damage [11]. The link between myocardial hypoxia and the development of heart failure has been speculated [12,13,14]. A recent study has demonstrated that gradual systemic hypoxemia can reduce reactive oxygen species (ROS) and oxidative DNA damage in cardiomyocytes, which is sufficient to induce cell cycle re-entry of adult cardiomyocytes [15]. The brain is one of the heaviest consumers of oxygen in the body. Insufficient oxygen supplements would lead to neuronal cell death [16,17,18,19] and structural and functional damage in the brain [20,21,22]. Furthermore, the liver also has a unique anatomical structure and acts as a master metabolic organ [23]. Maintenance of liver function requires a considerable amount of oxygen [24]. Previous studies have revealed that the pathogenesis of several hepatic diseases is related to hypoxia [25,26]. The kidney is a high-blood flow and high-perfused organ, and the low oxygen supply and high oxygen demand make the kidney vulnerable to hypoxia [27]. The regulation of erythropoietin, which is mainly produced by the kidney, is part of the classical physiological response to hypoxia [9]. In addition, exposure to high-altitude hypoxia increases the incidence of kidney disease [28,29,30]. The structural and functional alterations of these organs must have a unique molecular and metabolic basis. In fact, it is well known that molecular changes induced by hypoxia-inducible factors (HIFs) are associated with physiological and pathological responses to hypoxia [31]. Energy metabolism has important implications for organ function. The HIF-1α protein is present in the brain, kidney, liver and heart of mice kept under normoxic conditions and dramatically increased in these organs in response to short-term hypoxia, with an organ-specific regulation [32]. However, after long-term hypoxic exposure, HIF-1α becomes undetectable in the heart and liver, although it is highly expressed in the brain and kidney. Moreover, apoptosis was marked in the heart, slightly in the kidney medulla, and undetectable in other organs [33,34]. Based on the above data, we suggest that by inducing different metabolic changes, hypoxia may exert different effects on the heart, brain, liver and kidney.

When the oxygen supply of a tissue is restricted, mitochondria emit signals to generate ROS [31], which in turn stabilizes oxygen-sensing HIFs. Among the three isoforms of HIFs, only HIF-1α is found in all cell types and acts as a core regulator. HIF-1α also mediates primary transcriptional response to hypoxia [35,36,37]. Previous studies have demonstrated that the upregulation of HIF-1α leads to a shift in cell metabolism under hypoxic conditions [38,39,40,41], and metabolic disorders are involved in the development of hypoxic diseases [37,42,43]. HIF-1α activates the expression of genes that encode glucose transporters and glycolytic enzymes, thereby increasing cell glucose utilization and glycolytic flux [44,45,46]. Glycolysis then provides a path for ATP production at low O_2_ tension. Furthermore, HIFs have been implicated in promoting lipogenesis by changing the carboxylation of glutamine to ketoglutarate [47,48]. However, the metabolic characteristics of vulnerable organs suffering from hypoxia remain unclear.

This study aimed to test the hypoxic sensitivity of visceral organs and to identify which organ is the most vulnerable to hypoxic damage. To this end, we applied in vivo and in vitro imaging of anti-HIF-1α-NMs-cy5.5 nanocomposites to determine the accumulation of HIF-1α in the body after acute hypoxia exposure. Furthermore, we used immunofluorescence and Western blotting to locate and quantify the expression of HIF-1α in the brain and visceral organs. To clarify the functions of acute hypoxic exposure, we performed nuclear magnetic resonance (NMR)-metabolomic profiling of the metabolic features of the liver, kidney and brain.

## 2. Results

### 2.1. Physiological and Behavioral Alterations

**Figure 1:**
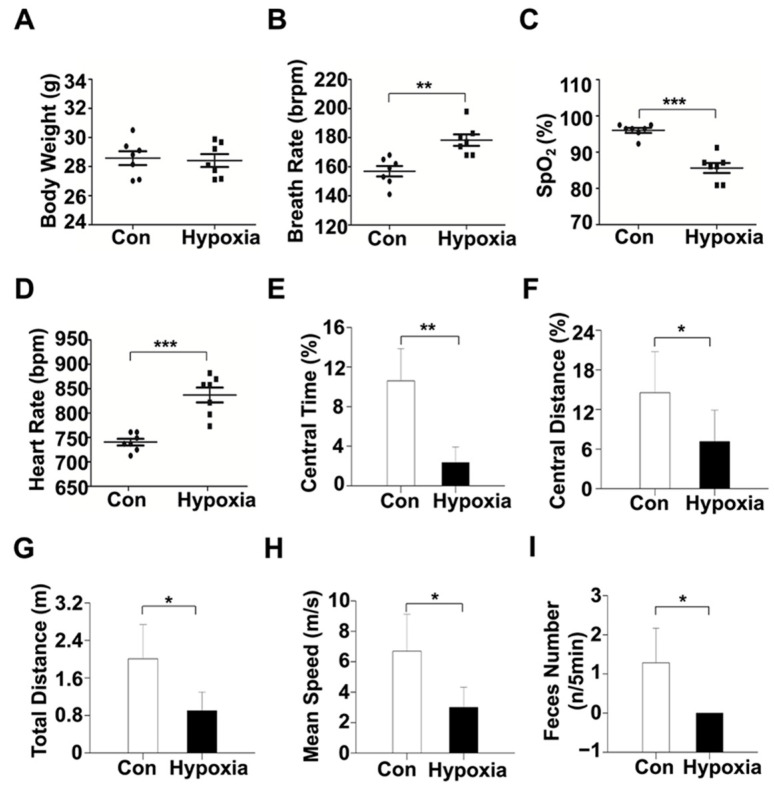
Changes of physiological values and behavior metrics in hypoxic mice. (**A**–**D**) Body weight, respiratory frequency, SpO_2_ and heart rate in hypoxic mice and controls (both, *n* = 7). (**E**–**I**) Open field test in hypoxic mice and controls. *, *p* < 0.05, **, *p* < 0.01, ***, *p* < 0.001. The error bars represent SEM.

Compared with control mice, hypoxic mice showed significantly decreased peripheral oxygen saturation (SpO_2_) and significantly increased respiratory frequency and heart rate (Figure 1A–D). In the open field test, both the number of grooming and percent of time spent in the center of the open field, the total distance and mean locomotor speed were significantly lower in hypoxic mice compared with controls (Figure 1E–I).

### 2.2. HIF-1α Expressions in the Liver, Kidney and Brain After Acute Hypoxic Exposure

**Figure 2:**
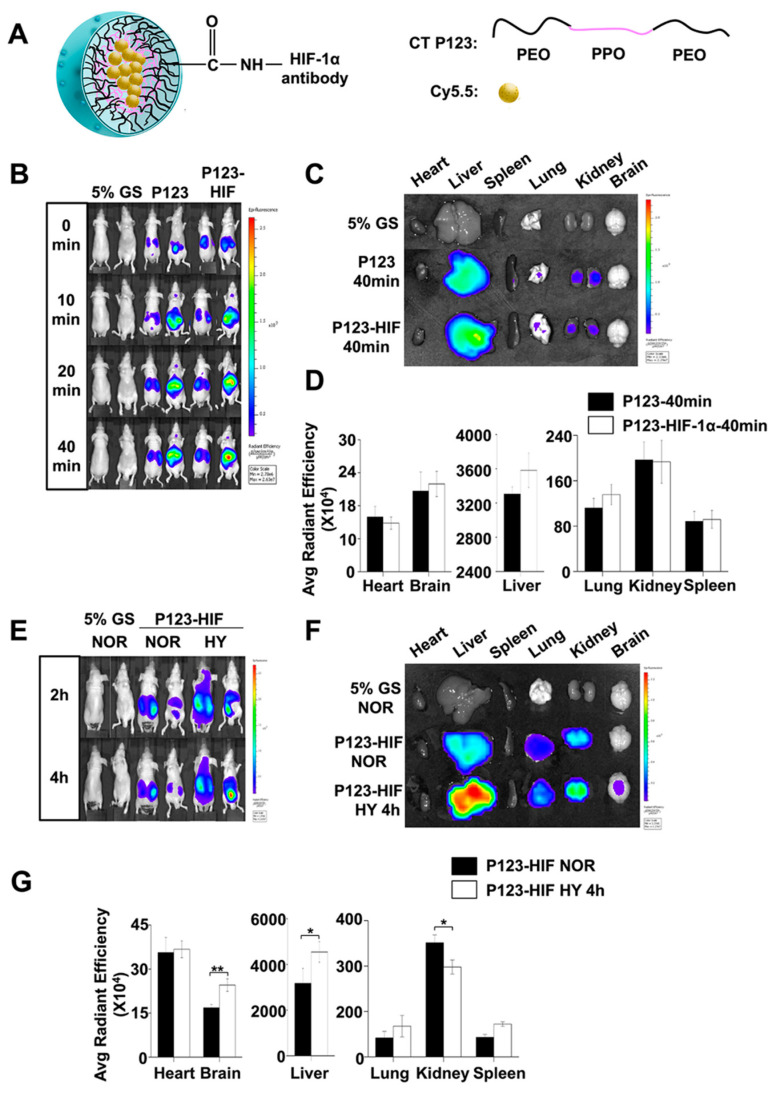
In vivo and in vitro fluorescence imaging of HIF-1α accumulation after hypoxic exposure. (**A**) Schematic representation of the synthesis of Pluronic P123 copolymer and strategy of modification of HIF-1α antibody. The left figure shows the structure of the nanocomposite of anti-HIF-1α-NMs-cy5.5; the right figure displays the carboxylic acid on the terminal end of the PEO which is conjugated to anti-HIF-1α by carbodiimide coupling chemistry. CT P123, carboxyl-terminated Pluronic P123 nano micelles; PEO, poly (ethylene oxide); PPO, poly (propylene oxide). (**B**) The metabolic kinetics of the nanocomposites in vivo was first tested before the hypoxia experiment. Fluorescence images were taken at 0, 10, 20 and 40 min after injection of anti-HIF-1α-NMs-cy5.5. After injection for 40 min, a strong and stable near-infrared fluorescence signal was observed. Mice injected with of glucose solution (GS) and NMs-cy5.5 (P123) served as controls. (**C**) In vitro fluorescence images of the organs excised from body 40 min after injection of nanocomposites. (**D**) Quantification of the nano-carriers accumulated in different organs (*n* = 3). (**E**) In vivo fluorescence imaging was performed in nude mice when strong and stable NIRF signal was observed. GS group: mice were injected with 5% glucose solution; NOR group: mice were injected with P123-HIF (anti-HIF-1α-NMs-cy5.5 nanocomposites) under normoxia condition; HY group: mice were exposed to 8% O2. (**F**) In vitro fluorescence images of the organs 4 h after hypoxia exposure. (**G**) Quantification of the nano-carriers accumulated in different organs after hypoxia (*n* = 3). *, *p* < 0.05, **, *p* < 0.01. The error bars represent SEM.

According to our previous report, anti-HIF-1α antibody-conjugated Pluronic P123 micelles can target HIF-1α in vivo [49]. When mixed with cy5.5, it can be self-organized into a single molecular micelle with the core of cy5.5, forming the nanocomposites of anti-HIF-1α-NMs-cy5.5. To determine the real-time biodistribution of the HIF-1α protein expression, we injected anti-HIF-1α-NMs-cy5.5 intravenously into nude mice, and analyzed the time-dependent distribution of the nanocomposites using in vivo fluorescence imaging (Figure 2A–F). A strong near-infrared fluorescence (NIRF) signal was observed in the whole body immediately after injection, and then it was gradually increased as the time elapsed until 40 min after injection (Figure 2B). No significant differences were observed in the distribution of fluorescence signal in anti-HIF-1α-NMs-cy5.5 all over the body compared with anti-HIF-1α antibody unconjugated nanocomposite (Figure 2C,D). Time-dependent strong fluorescence signals of anti-HIF-1α-NMs-cy5.5 were detected in the liver, kidney and brain in hypoxic mice compared with control mice (Figure 2E). For more accurate measurement, brain and major visceral organs (heart, liver, spleen, lung and kidney) were removed 4 h after hypoxia and analyzed directly on the fluorescent imager. The liver, kidney and brain showed marked accumulations of anti-HIF-1α-NMs-cy5.5 in the hypoxic mice compared with control mice (Figure 2F,G).

**Figure 3:**
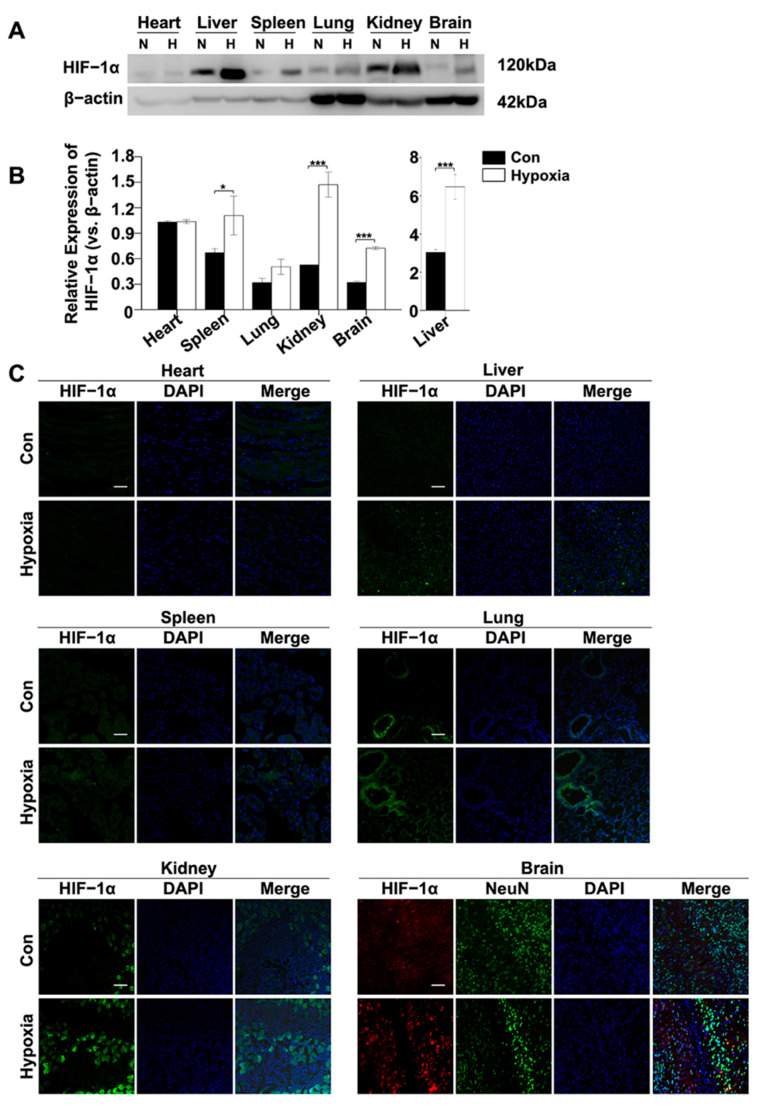
Locations and quantifications of HIF-1α protein in organs. (**A**) Western blot shows HIF-1α protein concentrations in the tissues of hypoxic mice (H) and control mice (N). (**B**) Statistic of HIF-1α protein levels. *, *p* < 0.05, ***, *p* < 0.001. (**C**) Locations of HIF-1α expressions in organs. Scale bar, 50 μm.

Western blot analysis showed that HIF-1α protein expressions were significantly increased in the liver, kidney and brain in hypoxic mice compared with controls, with by far the highest expression in the liver (Figure 3A,B).

### 2.3. Locations of HIF-1α Protein in the Liver, Kidney and Brain of Hypoxic Mice

In the liver, HIF-1α protein was expressed in the nuclear of hepatocytes, but not in the zonal and endothelial cells of the central and portal veins. In the kidney, the HIF-1α protein was discovered in the endothelial cells of distal convoluted tubules, but not in the glomerular endothelial and vascular endothelial cells. In the brain, HIF-1α protein was expressed in the neurons of the cerebral cortex of the brain in mice. In the heart and spleen, HIF-1α protein was not detected (Figure 3C). Furthermore, the expression level of HIF-1α was low in bronchial endothelial cells of the lung; however, there was no significant difference existed between hypoxic mice and controls (Figure 3C).

### 2.4. Metabolic Features in the Liver

**Figure 4:**
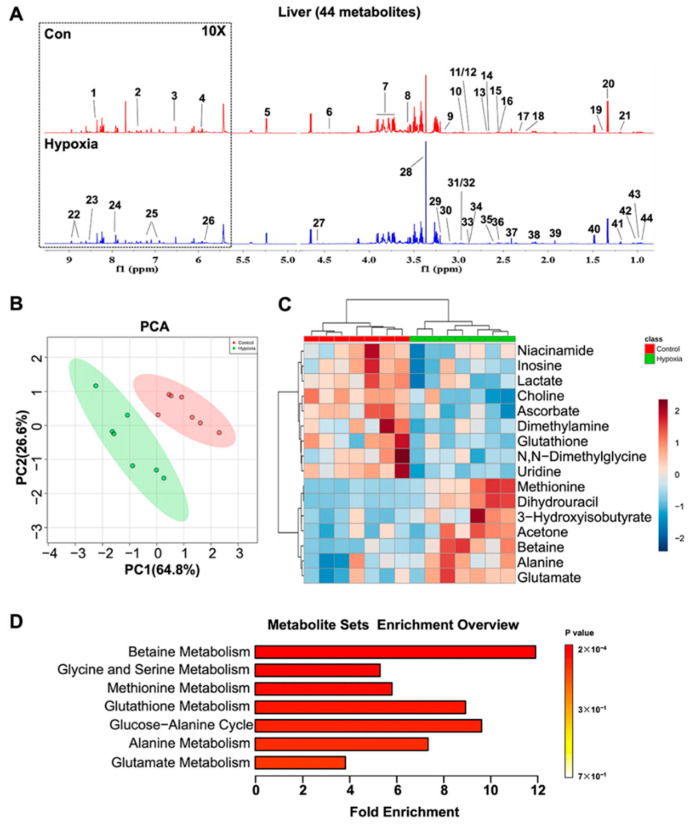
Metabolomic analysis of the liver. (**A**) Representative 1D ^1^H NMR spectra of aqueous extracts derived from the liver. 1, Inosine; 2, Phenylalanine; 3, Fumarate; 4, Uridine; 5, Mannose; 6, Glycerophosphocholine; 7, Glucose; 8, Glycine; 9, β-Alanine; 10, N,N-Dimethylglycine; 11, Asparagine; 12, Aspartate; 13, Sarcosine; 14, Dimethylamine; 15, Malate; 16, Dihydrouracil; 17, Glutamate; 18, Acetone; 19, 2-Phenylpropionate; 20, Lactate; 21, 3-Hydroxyisobutyrate; 22, Niacinamide; 23, Adenosine 5’-monophosphate; 24, Histamine; 25, Tyrosine; 26, Uracil; 27, Ascorbate; 28, Betaine; 29, Choline; 30, Ornithine; 31, N-Methylhydantoin; 32, Creatine phosphate; 33, Methylguanidine; 34, Trimethylamine; 35, Methionine; 36, Glutathione; 37, Succinate; 38, Glutamine; 39, Acetate; 40, Alanine; 41, Ethanol; 42, Valine; 43, Leucine; 44, Isoleucine. (**B**) PCA score plot, illustrating a metabolic difference between hypoxic mice and controls. The *x*-axis represents the first PC accounting for 64.8% of the total variation, and the *y*-axis denotes the second PC accounting for 26.6% of the total variation. Ovals in the panel highlight the metabolic distinction between two groups. (**C**) Clustered heatmap plot of relative metabolite levels, showing metabolic derangement. (**D**) Significantly altered metabolic pathways.

Forty-four metabolites of liver were unambiguously identified based on the NMR spectra (Figure 4A). The PCA demonstrated that hypoxic mice displayed a distinctly different metabolic profile from control group (Figure 4B). Based on the relative integrals calculated from the 1D NMR spectra of aqueous liver extracts, the relative levels of the metabolites were quantified (Supplementary Table S1). Sixteen metabolites were significantly changed in hypoxic mice compared with controls, with seven metabolites (3-hydroxyisobutyrate, alanine, betaine, glutamate, acetone, dihydrouracil and methionine) upregulated and nine metabolites (choline, dimethylamine, glutathione, inosine, lactate, niacinamide, N,N-dimethylglycine, ascorbate and uridine) down-regulated (Figure 4C). These changed metabolites mostly were mainly nucleotide and amino acids. Furthermore, metabolite set enrichment analysis (MSEA) identified seven significantly altered metabolic pathways, involving in the metabolisms of betaine, glycine, serine, methionine, glutathione, glucose-alanine cycle, alanine and glutamate (Figure 4D). The glycine, serine, and threonine metabolic pathways were the most significantly disturbed (Supplementary Figure S2A).

### 2.5. Metabolic Features in the Kidney

**Figure 5:**
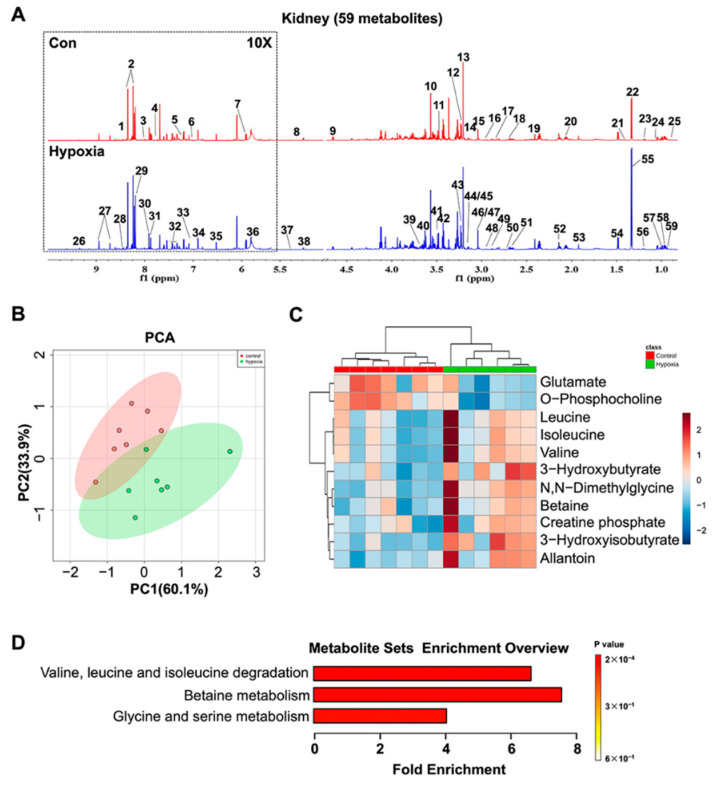
Metabolomic analysis of the kidney. (**A**) Representative 1D ^1^H NMR spectra of aqueous extracts derived from the kidney. 1, Inosinic acid; 2, Inosine; 3, 3-Methylxanthine; 4, Tryptophan; 5, Imidazole; 6, Anserine; 7, Uridine; 8, Glucuronate; 9, Glucose; 10, Glycine; 11, Methanol; 12, Betaine; 13, Choline; 14, Ethanolamine; 15, N,N-Dimethylformamide; 16, N-Methylhydantoin; 17, Asparagine; 18, Aspartate; 19, Succinate; 20, Glutamate; 21, 2-Phenylpropionate; 22, Threonine; 23, 3-Hydroxyisobutyrate; 24, 3-Hydroxybutyrate; 25, Pantothenate; 2, Nicotinamide adenine dinucleotide; 27, Niacinamide; 28, Formate; 29, Adenine; 30, Xanthine; 31, 4-Pyridoxate; 32, Phenylalanine; 33, Histamine; 34, Tyrosine; 35, Fumarate; 36, Uracil; 37, Allantoin; 38, Mannose; 39, Glycerol; 40, Myo-inositol; 41, Taurine; 42, O-Phosphocholine; 43, Glycerophosphocholine; 44, Malonate; 45, Cystine; 46, Creatine phosphate; 47, Creatinine; 48, N,N-Dimethylglycine; 49, Trimethylamine; 50, Dimethylamine; 51, 5,6-Dihydrouracil; 52, Methionine; 53, Acetate; 54, Alanine; 55, Lactate; 56, Ethanol; 57, Valine; 58, Leucine; 59, Isoleucine. (**B**) PCA score plot, illustrating the metabolic difference between hypoxic mice and controls. The *x*-axis represents the first PC accounting for 60.1% of the total variation, and the *y*-axis denotes the second PC accounting for 33.9% of the total variation. Ovals in the panel highlight the metabolic distinction between two groups. (**C**) Clustered heatmap plot of relative metabolite levels, showing the metabolic derangement. (**D**) Significantly altered metabolitc pathways.

Fifty-nine metabolites were unambiguously identified based on the NMR spectra (Figure 5A). The PCA demonstrated that hypoxic mice displayed a distinctly different metabolic profile from normoxic mice (Figure 5B). Pairwise PLS-DA analysis further illustrated that hypoxic mice were metabolically discriminated from controls (Supplementary Figure S1A). The validation plots of the corresponding RPTs indicated that the PLS-DA model was reliable (Supplementary Figure S1B). Based on the relative integrals calculated from the 1D NMR spectra of aqueous kidney extracts, the relative levels of the metabolites were quantified (Supplementary Table S2). Eleven metabolites were significantly changed in hypoxic mice compared with controls, with glutamate, betaine and o-phosphocholine down-regulated and valine, 3-hydroxyisobutyrate, 3-hydroxybutyrate, N,N-Dimethylglycine, allantoin, leucine and isoleucine upregulated (Figure 5D). MSEA identified three distinctly altered metabolic pathways, involving in the metabolisms of valine, leucine, isoleucine, betaine, glycine and serine (Figure 5D). The valine, leucine and isoleucine metabolic pathways were the most altered. (Supplementary Figure S2B).

### 2.6. Metabolic Features in the Brain

**Figure 6:**
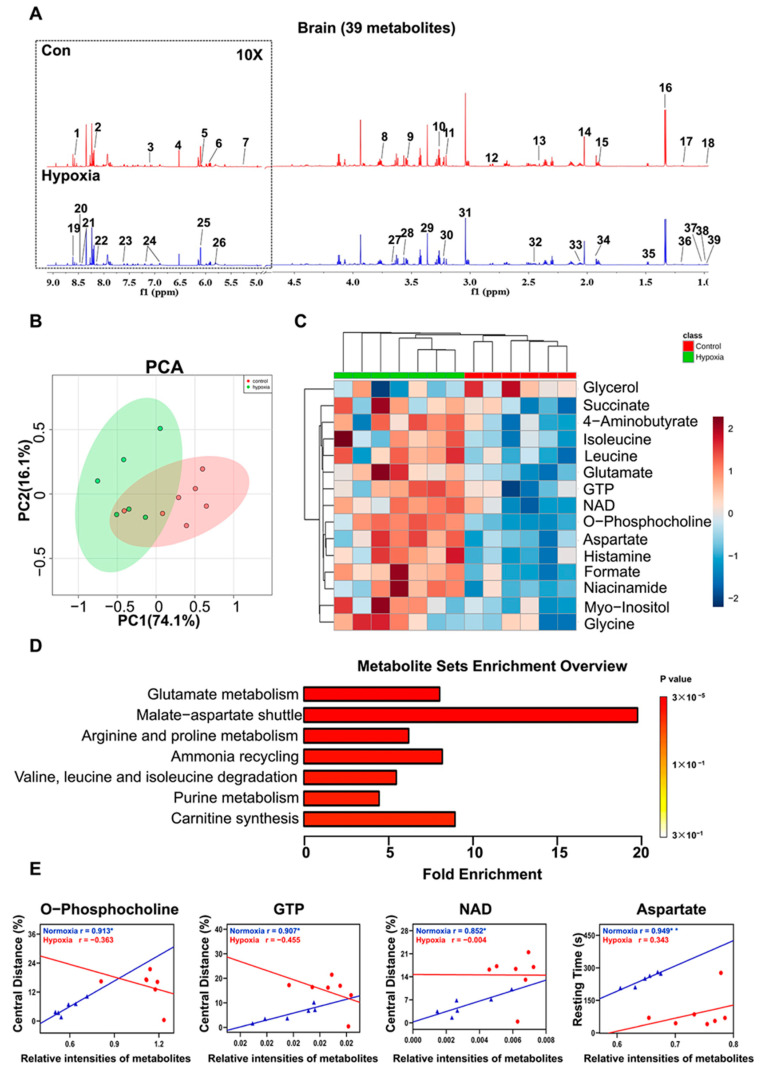
Metabolomic analysis of the brain. (**A**) Representative 1D ^1^H NMR spectra of aqueous extracts derived from the brain of mice. 1, Inosinic acid; 2, Adenine; 3, Histamine; 4, Fumarate; 5, Adenosine; 6, Uridine; 7, Glucose; 8, Ascorbate; 9, Myo-inositol; 10, Taurine; 11, O-Phosphocholine; 12, Aspartate; 13, Succinate; 14, N-Acetylaspartate; 15, 4-Aminobutyrate; 16, Lactate; 17, Ethanol; 18, 3-Hydroxyisobutyrate; 19, Adenosine monophosphate; 20, Formate; 21, Nicotinamide adenine dinucleotide; 22, Guanosine triphosphate; 23, Niacinamide; 24, Tyrosine; 25, Inosine; 26, Uracil; 27, Glycerol; 28, Glycine; 29, Caffeine; 30, Glycerophosphocholine; 31, Creatine phosphate; 32, Glutamine; 33, Glutamate; 34, Acetate; 35, Alanine; 36, 3-Hydroxyisobutyrate; 37, Valine; 38, Leucine; 39, Isoleucine. (**B**) PCA score plot, illustrating the distinct metabolic difference between hypoxic mice and controls. The *x*-axis represents the first PC accounting for 74.1% of the total variation, and the *y*-axis denotes the second PC accounting for 16.1% of the total variation. Ovals in the panel highlight the metabolic distinction between two groups. (**C**) Clustered heatmap plot of relative metabolite levels, showing metabolic derangement. (**D**) Significantly altered metabolic pathways. (**E**) The correlations of relative intensities of metabolites with mice performances in the open field test. *, *p* < 0.05.

Thirty-nine metabolites were unambiguously identified based on the NMR spectra (Figure 6A). The PCA demonstrated that hypoxic mice displayed a distinctly different metabolic profile from normoxic mice (Figure 6B). Pairwise PLS-DA analysis illustrated a distinct metabolic separation between hypoxic mice and controls (Supplementary Figure S1C). The validation plots of the corresponding RPTs confirmed that the PLS-DA model was valid (Supplementary Figure S1D). Based on the relative integrals calculated from the 1D NMR spectra of aqueous brain extracts, the relative levels of the metabolites were quantified (Supplementary Table S3). Fifteen metabolites were significantly changed in hypoxic mice compared with controls, with 4-aminobutyrate, glutamate, succinate, aspartate, o-phosphocholine, myo-inositol, glycine, guanosine triphosphate (GTP), histamine, formate, niacinamide, nicotinamide adenine dinucleotide (NAD), leucine and isoleucine upregulated and glycerol down-regulated. These changed metabolites mostly were carbohydrates and amino acids. MSEA identified seven distinctly altered metabolic pathways, involving in the metabolisms of glutamate, malate-aspartate shuttle, arginine, proline, ammonia recycling, valine, leucine and isoleucine degradation, purine and carnitine synthesis (Figure 6D). The alanine, aspartate and glutamate metabolic pathways were the most significantly altered (Supplementary Figure S2C).

In normoxic mice, the correlation analysis showed that the relative intensities of o-phosphocholine (r = 0.913, *p* = 0.011), GTP (r = 0.907, *p* = 0.013), NAD (r = 0.852, *p* = 0.031) were positively correlated with mice moving distance in the center floor in open field test, and the relative intensity of aspartate was positively correlated with resting time (r = 0.959, *p* = 0.004) (Figure 6E). No significant correlations were established between the relative intensities of the other metabolites and the behavior metrics in the hypoxia group.

## 3. Discussion

In this study, we investigated the effects of acute hypoxia on the brain and visceral organs by observing the metabolic characteristics and accumulation of oxygen-sensitive transcription factor HIF-1α. The expression levels of HIF-1α in the liver, kidney and brain were significantly higher than those in other organs after acute hypoxia exposure, with the highest expression levels in the liver. Unexpectedly, the heart did not show a higher expression of HIF-1α. Acute hypoxia exposure caused metabolic disorders in these organs with high HIF-1α expression, and the liver, kidney, and brain had distinct metabolic profiles but a consistent change in glutamate.

Two previous studies have observed HIF-1α distributions in the animal body at the mRNA level. The first study found that the exon I.1-derived HIF-1α mRNA isoform was differentially expressed in mice exposed to 7.5% O_2_, showing high levels in the kidney, tongue, stomach and testis, but undetectable in the liver, whereas the exon I.2 isoform displayed a ubiquitous expression pattern in all mouse tissues examined [50]. This study verified the total HIF-1α mRNA expression in the liver and found a gradual decrease in HIF-1α mRNA after prolonged hypoxia (7.5% O_2_ for up to 72 h). On the other hand, the second study showed significant differences in the expression of prolyl hydroxylase (PHD) mRNA in organs of rats exposed to 8% O_2_ for 8 h [51]. PHD can catalyze the hydroxylation reaction of HIF-1, and PHD2 is the dominant isoform in regulating HIF detectable in all organs, with the highest levels in the heart. PHD2 upregulation could potentially lead to attenuated HIF accumulation in the heart. This finding in PHD2 supports the results of the present study, which showed low HIF-1α protein expression in the heart. HIF-1α protein expression has been directly observed in mice subjected to acute hypoxia (1–12 h) [32], and higher expression of HIF-1α was found in the brain, kidney, liver, heart and skeletal muscle; however, HIF-1α in the liver and kidney reached maximum levels after 1 h and disappeared after 4 h after hypoxia. These findings were inconsistent with our results and the findings of Bianciardi et al. [33], because we still observed higher concentrations of HIF-1α in the liver and kidney of mice 6 h after hypoxia, while Bianciardi et al. found that HIF-1α was expressed in the kidneys of rats exposed to chronic hypoxia (10% O_2_) for 2 weeks. Another inconsistency was that HIF-1α protein was detected in the heart, but not in our study [33,51]. In summary, the different expression of HIF-1α in the above studies mainly exists in the heart and liver, showing HIF-1α expression in the heart [32,50], and liver [32,51], showing HIF-1α expression in the two organs, but not in other studies [33]. It remains to be elucidated whether this is due to the faster return of HIF-1α to baseline levels in these two organs. In our study, in vivo imaging could determine the real-time biodistribution of HIF-1α protein concentration in the whole body with a high targeting ability, as this method avoids the degradation of HIF-1α.

**Figure 7:**
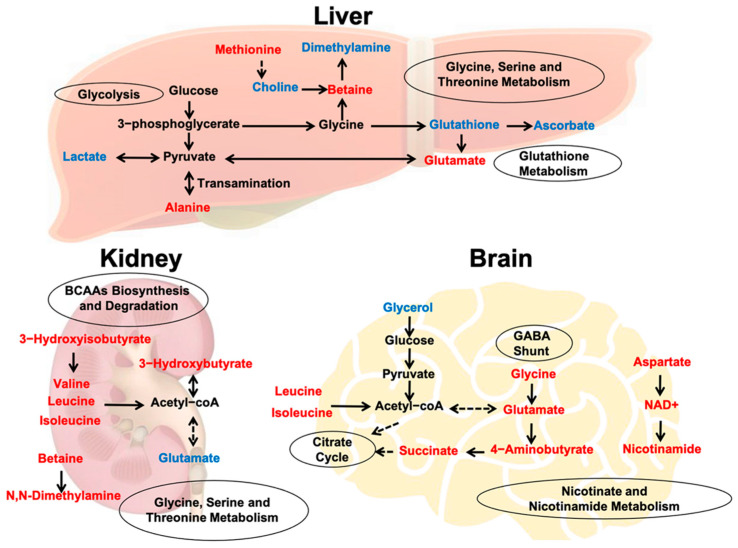
Overview of hypoxia altered metabolic pathways. Blue indicates the down-regulated metabolites, whereas red indicates upregulated metabolites. Hypoxic exposure significantly altered amino acid metabolism and energy metabolism, with glutamate changed in all the liver, kidney and brain.

Compared with the kidney and brain, the hypoxic mice displayed remarkably more altered metabolites in the liver. As the central metabolic organ that maintains ammonia metabolism and blood glucose levels, the liver has a multitude of functions that require a considerable amount of oxygen [24,52]. The predominant metabolic changes in the liver might indicate that hepatic metabolic disorders are more closely related to acute hypoxia exposure. Additionally, we found that HIF-1α was mostly expressed in the liver after hypoxia. HIFs regulate major metabolic liver functions [53], which is consistent with the results of hepatic metabolic disorders. Furthermore, the liver is the major organ involved in gluconeogenesis [54], and the abnormal expression of HIF-1α is related to impaired gluconeogenesis during liver regeneration [55]. The different metabolic responses and mechanisms in the liver remain relatively unknown during acute hypoxia exposure. The importance of oxygen as a regulator of carbohydrate metabolism has been confirmed in rat liver studies [56]. During hypoxic exposure, animals switch from oxidative phosphorylation to glycolysis. Glucose is processed via glycolysis to pyruvate with an ATP yield. Then, pyruvate is either directly converted to lactate or reacts with glutamate in a transamination reaction, yielding alanine and α-ketoglutarate. In the present study, we observed significantly decreased levels of lactate and increased levels of alanine and glutamate after acute hypoxia (Figure 7), suggesting that pyruvate conversion pathways were shifted towards alanine in the liver.

On the other hand, glutamate levels can be increased by glutathione metabolism. Glutathione is the main cellular antioxidant in cells that efficiently counteracts oxidative stress [57]. As a well-known antioxidant, ascorbate can reduce ROS content in hepatocyte mitochondria. Moreover, we observed gradually decreased levels glutathione and ascorbate, suggesting a partially weakened antioxidant defense system after acute hypoxia exposure. Furthermore, betaine, which is derived from choline via an irreversible oxidation reaction, functions as an osmolyte capable of stabilizing cell membranes and exerts antioxidant effects by increasing the levels of methionine [58,59]. Therefore, the increased methionine and betaine levels and decreased choline in the hypoxic liver suggest that acute hypoxic stress increases liver cell membrane permeability and promotes betaine biosynthesis.

In our study, 11 differential metabolites were significantly altered in the kidneys of hypoxic mice, including increased branched chain amino acids (BCAA) such as valine, leucine, isoleucine and 3-hydroxyisobutyrate (3-HIBA). 3-HIBA is a catabolic valine intermediate. BCAAs are not only involved in protein synthesis, but also play an important role in glucose metabolism. A previous study has shown that BCAAs such as leucine, valine, and isoleucine are involved in the treatment of chronic renal failure [60]. High concentrations of BCAAs have also been observed in several pathophysiological conditions [61,62]. In acute hypoxic conditions in our study, mitochondrial dysfunction resulted in a restrictive utilization of BCAAs, which gave rise to the accumulation of BCAAs in the kidney. We observed that betaine and N,N-dimethylglycine were increased in the kidneys of hypoxic mice. Betaine is utilized in the kidney primarily as an osmoprotectant [63], and the inflammation caused by hypoxia can be diminished by betaine [64]. Thus, increased betaine indicates that it may act as both an osmoprotectant and an antioxidant after hypoxia exposure. In addition, as a structural component in the membrane, phosphocholine is decreased after hypoxia [65], suggesting that acute hypoxic stress increases kidney cell membrane permeability and decreases integrity. Additionally, allantoin is a product of uric acid oxidation [66], and its elevated level after hypoxia indicates that hypoxia might increase the attack of highly reactive ROS on uric acid. In summary, these altered metabolites in the kidneys might protect against acute hypoxic stress.

Hypoxia has been shown to induce cognitive deficits [67,68]. In our study, we observed an anxiety-like behavior in mice exposed to hypoxia for several hours. Furthermore, 15 differential metabolites were found in the hypoxic mouse brain, and significant correlations existed between aspartate, O-phosphocholine, GTP, NAD, and behavior metrics in the normoxic group. However, these relationships were disrupted in the hypoxia group. O-phosphocholine is an early cord metabolite index and is an outcome of hypoxic-ischemic encephalopathy [69]. Both NAD and NADPH play important roles in neuroprotection [70] and are regulated by HIF-1α to maintain cellular redox homeostasis and energy metabolism [71]. Aspartic acid and glutamic acid are excitatory amino acid neurotransmitters [72]. The breakdown of these correlations suggests that hypoxia stress promotes the release of excitatory neurotransmitters and changes energy metabolism. Moreover, BCAAs play critical roles in neurotransmitter synthesis in the brain [73]. We observed that leucine and isoleucine levels were significantly increased after hypoxia, which was in accordance with the increase in excitatory neurotransmitters. Additionally, the GABA shunt is an alternative energy production pathway activated during cellular stress, which can be triggered by local hypoperfusion and subsequent hypoxia [74]. Our results were consistent with those observed in a previous study, indicating that GABA shunt was profoundly promoted after hypoxia. Glycerol was also thought to be a marker of cell membrane degradation [75]; however, we observed a decrease in glycerol after hypoxia, suggesting that acute hypoxia stress did not affect the integrity of the cell membrane in the brain. In summary, the cognitive deficits caused by acute hypoxia stress are mostly associated with energy metabolism abnormalities and increases in some neurotransmitters.

Distinct metabolic features of the liver, kidney, and brain in hypoxic mice may be due to the different physiological functions of these organs. In our study, changes in glutamate levels were consistently detected in the liver, kidney, and brain after acute hypoxia exposure. It is well known that glutamate is a potential survival factor that enhances cell survival in hypoxia/reoxygenation injury [76,77]. Glutamate is the main excitatory neurotransmitter [78] in the brain and is released by hypoxia-activated microglia [79,80]. In the kidney, EAAC1 is the only cloned glutamate transporter that plays an important role in glutamate control [81]. A significantly decreased level of glutamate was found in the kidney after hypoxia, which might be a possible consequence of enhanced EAAC1 production [82]. In the liver, the increased level of glutamate suggests that mitochondrial glucose metabolism is reduced under hypoxia, and an exogenous supply of glutamine could fuel the Krebs cycle and contribute to the glutamate pool in hepatocytes to preserve liver bioenergetics [83]. Additionally, hypoxia regulates glutamic acid and decarboxylase enzyme levels [84]. In summary, glutamate release in the liver, kidney, and brain might indicate an enhanced metabolic utilization and a protective effect against hypoxia.

In conclusion, our study showed a higher concentration of HIF-1α in the liver, kidney and brain after acute hypoxia exposure, which is partially consistent with the findings of previous studies. Significantly, our study revealed that the liver had the highest levels of HIF-1α among visceral organs. Moreover, the liver, kidney, and brain may be the most vulnerable to hypoxic damage. Interestingly, the hypoxic mice displayed considerably more altered metabolites in the liver. The liver, kidney, and brain have distinct metabolic profiles, except for their identical glutamate changes. Our study expands the understanding of the molecular mechanisms underlying hypoxia and may contribute to the treatment of hypoxic injury associated with clinical diseases.

## 4. Materials and Methods

### 4.1. Animals and Patterns of Hypoxic Exposure

A total of 14 Kunming male mice (7 months) and 14 BALB/c male nude mice (7 weeks) were purchased from the experimental animal center of Xiamen University. According to a previous study that HIF-1α proteins were only expressed in the brain, kidney, liver and heart of mice exposed to very low oxygen concentration (6% O_2_) within several hours [32], and therefore, in this study mice were exposed to oxygen at this concentration. Kunming mice were exposed to 6% O_2_ in a gas control chamber for 6 h for physiological measurements, behavioral tests, Western blot analysis and immunofluorescence staining of HIF-1α expression in tissues and NMR-based metabolomics study. As nude mice were hard to survive extremely low oxygen, they were exposed to a relatively higher level of oxygen at 8% O_2_ for 4 h in the study of in vivo imaging of HIF-1α accumulation. Control mice were raised in room air conditions. All mice were maintained under standard conditions at 22 ± 1 °C, with free access to food and water, and exposed to a 12 h:12 h light/dark cycle. The protocols have been reviewed and approved by the committee on animal care and used at Xiamen University.

### 4.2. Physiological Measurements

Respiratory frequency, heart rate and SpO_2_ were measured using multi-parameter monitoring equipment (STARR multi-parameter monitor, RWD Life Science Co., Shen Zhen, China).

### 4.3. Open Field Test

The open field test was used to assess anxiety-like behavior. Immediately after hypoxic exposure, mice were taken out of the hypoxic chamber for the test in the open field. The apparatus was a square open-field box (60 × 60 × 50 cm). The floor was divided into 9 squares. The central square was regarded as the “center” of the floor. The test started by placing mice in the center of the field. The activities of each mouse within 5 min were video-tracked and counted, which included the total of center entry, defecation, grooming, rearing, moving distance and moving speed.

### 4.4. In Vivo and In Vitro Imaging of HIF-1α Accumulation

#### 4.4.1. Preparation of Anti-HIF-1α Antibody-Conjugated Pluronic P123 Micelles

The Pluronic block copolymers contained poly (ethylene oxide) (PEO) and poly (propylene oxide) (PPO), showing the structural form as PEO-PPO-PEO, with PEO as the water-soluble block. Carboxyl-terminated Pluronic P123 nano micelles were prepared using the method as described in our previous study [49]. The concrete steps were as follows: briefly, 0.5 mg cy5.5 and 2 mg carboxylate-functionalized copolymer Pluronic P123 were dissolved in methyl cyanides under the help of ultrasonic treatment, and dried in a rotary evaporator at reduced pressure to form a thin-film layer, which was resuspended in 1 mL ultrapure water containing 5% trehalose until completely hydrated. Then, the nano micelles dispersion was serially passed through 0.2 mm pore size filters (Millipore) and the insoluble substance was removed from the solution. Final yields of carboxyl-terminated Pluronic P123 nano micelles filled with cy5.5 were obtained. Finally, the cy5.5-loaded Pluronic NMs were incubated with 400 mM 1-ethyl-3-(3-dimethylaminopropyl)-carbodiimide and 100 mM N-hydroxysuccinimide for 15 min at room temperature with gentle stirring. After Pluronic P123 micelles were prepared, they were covalently linked to HIF-1α monoclonal antibody (1% weight compared with polymer concentration) for further experiments.

#### 4.4.2. Biodistribution of HIF-1α Protein Using In Vivo Imaging

Nude mice were anesthetized with isoflurane. For in vivo study, the nanocomposites (anti-HIF-1α-antibody-conjugated P123 micelles encapsulated with cy5.5) were injected into nude mice by intravenous route. This study included two steps: (1) Metabolic kinetics of the nanocomposites in vivo was first tested before the hypoxia experiment. Nude mice, which were injected into 5% glucose solution or anti-HIF-1α antibody unconjugated nanocomposite, were served as controls. Fluorescence images were taken by the IVIS Spectrum imaging system (Caliper Life Science, Hopkinton, MA, USA) at 0, 10, 20 and 40 min after injection. In this study, we observed that the strongest and stable NIRF signal appeared at time-point of 40 min after injection. Therefore, in the next hypoxia experiment, after injection for 40 min, mice were exposed to 8% O_2_ hypoxia. (2) Hypoxia experiment. Normoxic nude mice were set as controls. The images were taken at 2 and 4 h after hypoxia. After the last in vivo fluorescence imaging, brain and visceral organs were excised from mice body for in vitro imaging study.

### 4.5. Western Blot

Mice were sacrificed immediately (<5 s) after being taken out of the hypoxic chamber. Organs were homogenized and solubilized in RIPA lysis buffer with the protease inhibitor cocktail (Roche, Basel, Switzerland). Tissue homogenates were thereby sonicated for 1 min and centrifuged at 13000× *g* for 15 min at 4 °C. The supernatants were collected, and protein concentrations were determined by BCA Protein Assay Kit (Thermo Fisher Scientific, Waltham, MA, USA). Equal amounts of proteins were subjected to SDS-PAGE and transferred to PVDF membranes for immunoblotting analysis. The membranes were first blocked with fat-free 5% milk in Tris-buffered saline with 0.1% Tween 20 (TBST), and then successively incubated with primary antibodies (HIF-1α: ABclonal Biotech Co., Ltd., Cambridge, MA, USA; actin: Cell Signaling Technology, Beverly, MA, USA) and secondary antibodies. Secondary antibodies included anti-rabbit and anti-mouse antibodies (Bio-Rad, Laboratories, Inc., Shanghai, China). Finally, blots were visualized by enhanced chemiluminescence reagents (Thermo Fisher Scientific, San Jose, CA, USA). Actin served as an internal control.

### 4.6. Immunofluorescence Staining

Mice were sacrificed immediately (<5 s) after being taken out of the hypoxic chamber. The heart, liver, spleen, lung, kidney and brain were immediately frozen in liquid nitrogen, and stored at −80 °C. Sections (20 μm) of the tissues were cut by using a cryostat microtome (HM 505-E, Germany). After 4% paraformaldehyde fixed for 10 min, sections were incubated with HIF-1α rabbit monoclonal antibody (1:500 dilution, Abcam, Cambridge, MA, USA) in PBST containing 0.3% Triton X-100 overnight at 4 °C. For brain, sections were incubated with both HIF-1α antibody and Alexa Fluor 488 conjugate mouse anti-NeuN monoclonal antibody (1:200 dilution, Merck Millipore, Darmstadt, Germany) at the same time. Then, the sections were incubated with 10% donkey serum for 20 min. Subsequently, sections were incubated with the specific donkey anti-rabbit immunoglobulin G (IgG) (FITC-labeled or Tetramethylrhodamine (TRITC)-labeled, 1:200 dilution, Santa Cruz Biotechnology, Dallas, TX, USA) for 2 h at room temperature. Finally, the sections were covered with glycerol and positive cells were observed using a laser scanning confocal microscope.

### 4.7. NMR Sample Preparation and Measurements

Mice were sacrificed immediately (<5 s) after being taken out of the hypoxic chamber. Aqueous intracellular metabolites were extracted from the liver, kidney and brain for the NMR analysis according to our protocol described previously [85]. Generally, the liver, kidney and brain samples were homogenized after adding ice-cold methanol, chloroform and water at a volume ratio of 4:4:2.85 to obtain a two-phase extract. Only the upper polar tissue extracts were lyophilized and suspended in 550 μL of NMR buffer (50 mM sodium phosphate buffer, pH 7.4, in D_2_O) using 0.1 mM sodium 3- (trimethylsilyl) propionate-2,2,3,3-d4 (TSP). D_2_O was used for field-frequency lock, and TSP was used to provide the chemical shift reference (δ 0.00). All the samples were vortexed uniformly and centrifuged (12000× *g* for 10 min at 4 °C), and finally transferred into 5-mm NMR tubes.

NMR experiments were performed on a Bruker Avance III 850 MHz spectrometer equipped with a high-sensitivity TCI cryoprobe (Bruker BioSpin, Rheinstetten, Germany). One dimensional (1D) 1H spectra was recorded on aqueous extracts of tissue using the pulse sequence NOESYGPPR1D [RD–G1–90°–t1–90°–τ_m_–G2–90°–ACQ] with water suppression during the relaxation delay and mixing time. The parameters were set as follows: relaxation delay 4 s, short delay 4 μs and mixing time 10 ms. Pulsed gradients G1 and G2 were used to improve water suppression quality. A total of 32 transients were collected into 64 K data points using a spectral width of 17 KHz with an acquisition time (ACQ) of 1.88 s.

The MestReNova software (Version 9.0, Mestrelab Research S. L, Spain) was used to process NMR spectral data. To reduce concentration differences among the samples, NMR spectral integrals of metabolites were normalized to the total spectral integrals by using MatLab (Version 2011b; Math Works Inc., Natick, MA, USA). Based on the NMR spectra, metabolites were identified by a combination of the Chenomx NMR Suite (Version 8.2, Chenomx Inc., Edmonton, AB, Canada), the Human Metabolome Data Base (HMDB, http://www.hmdb.ca/ accessed on 31 March 2021) and relevant published references. Multivariate statistical analysis was conducted using the SIMCA-P + software (Version 12.0.1, Umetrics AB, Umea, Sweden).

For multivariate data analysis, the normalized spectral data were imported into the SIMCA-P software (Version 12.0, Umetrics AB, Umea, Sweden), and scaled by Pareto scaling to increase the importance of low-level metabolites without significant amplification of noise. The principal component analysis (PCA) was performed to observe the grouping trends, highlight outliers and show clusters among the groups. MSEA were applied to identify the altered metabolic pathways in hypoxic mice (*p* < 0.05), using the module of metabolite sets enrichment analysis (MetaboAnalyst 4.0 webserver).

### 4.8. Statistical Analysis

All data were quantitatively analyzed by the SPSS16 software data program. Differences between the groups were pairwisely compared by using an independent samples t test, with *p* < 0.05 was considered statistically significant.

## Data Availability

All data generated or analyzed during this study are included in the manuscript or Supplementary Materials.

## Supplementary Materials

The following are available online at https://www.mdpi.com/article/10.3390/ijms22136705/s1, Figure S1: PLS-DA score plots and validation plots of 1D 1H NMR data for aqueous extracts derived from the kidney and brain of mice, Figure S2: Significantly altered metabolic pathways associated with the hypoxia mice relative to normoxic mice, Table S1: Comparison of metabolite levels between two groups of mice based on relative integrals calculated from the 1D 1H NMR spectra of liver, Table S2: Comparison of metabolite levels between two groups of mice based on relative integrals calculated from the 1D 1H NMR spectra of kidney, Table S3: Comparison of metabolite levels between two groups of mice based on relative integrals calculated from the 1D 1H NMR spectra of brain.
