# Supplementary material for: Different Expressions of HIF-1α and Metabolism in Brain and Major Visceral Organs of Acute Hypoxic Mice
Source: Int J Mol Sci. 2021 Jun 23;22(13):6705. doi: 10.3390/ijms22136705 (PMC8268807; doi:10.3390/ijms22136705)
Supplement: Supplementary file 1 [file ijms-22-06705-s001.zip › ijms-1186941-supplementary.pdf]

Supplementary Material

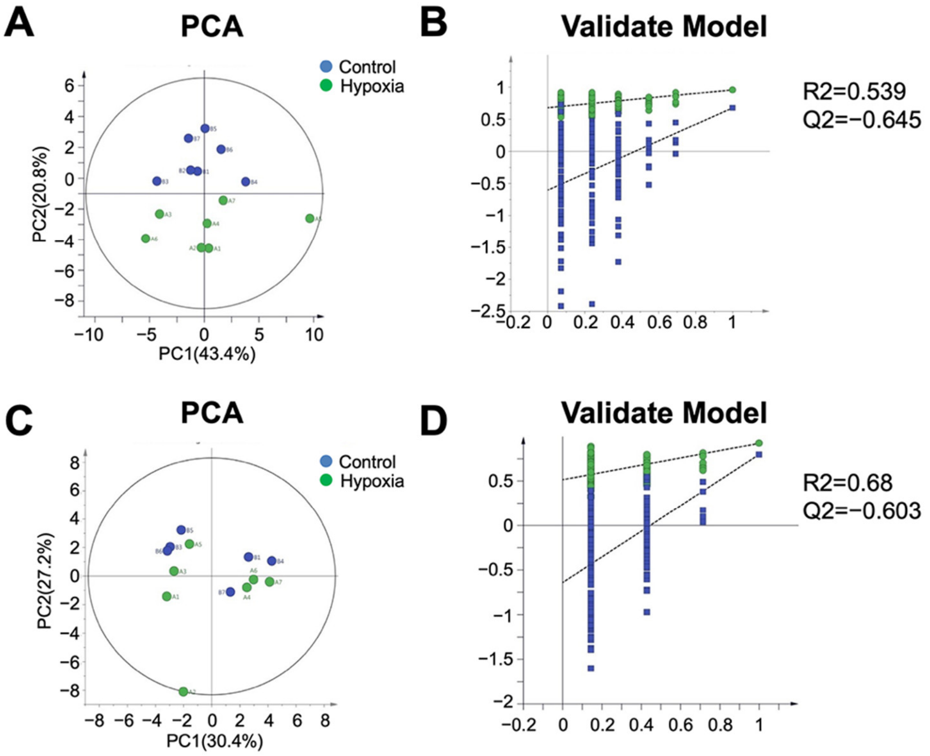

Supplementary Figure S1. PLS-DA score plots and validation plots of 1D ^{1}H NMR data for aqueous extracts derived from the kidney and brain of mice. (A, B) Hypoxia kidney *vs.* normoxic kidney; (C, D) Hypoxia brain *vs.* normoxic brain. The PLS-DA models were cross-validated to evaluate the robustness by a random permutation test (200 cycles). n=6-7 mice/group.

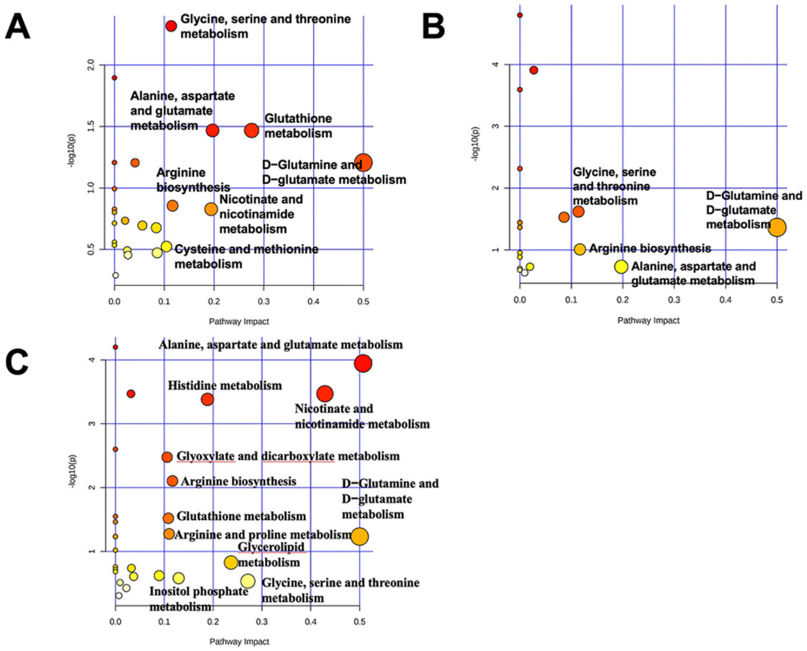

Supplementary Figure S2. Significantly altered metabolic pathways associated with the hypoxia mice relative to normoxic mice. (A) Hypoxia liver *vs.* normoxic liver; (B) Hypoxia kidney *vs.* normoxic kidney; (C) Hypoxia brain *vs.* normoxic brain. Based on the significant metabolites, significantly altered metabolic pathways were identified with p values < 0.005, using the Pathway Analysis module provided by MetaboAnalyst 4.0.

Supplementary Table S1. Comparison of metabolite levels between two groups of mice based on relative integrals calculated from the 1D ^{1}H NMR spectra of liver.

Metabolites	Mean ± standard deviation		Hypoxia
	Normoxic liver	Hypoxic liver	*vs.* Normoxia
3-Hydroxyisobutyrate	0.201±0.034	0.295±0.072	*
Acetate	0.512±0.118	0.519±0.1266	NS
Alanine	3.134±0.408	3.612±0.314	*
Asparagine	0.225±0.029	0.220±0.026	NS
Aspartate	0.089±0.016	0.090±0.015	NS
Betaine	1.372±0.217	2.219±0.617	*
Choline	2.183±0.214	1.443±0.405	**
Creatine phosphate	0.354±0.059	0.363±0.041	NS
Dimethylamine	0.078±0.010	0.068±0.005	*
Ethanol	0.512±0.132	0.594±0.410	NS
Fumarate	0.119±0.029	0.135±0.034	NS
Glucose	4.493±1.335	5.442±0.967	NS
Glutamate	1.149±0.213	1.481±0.206	*
Glutamine	1.761±0.234	1.932±0.312	NS
Glutathione	0.587±0.158	0.404±0.073	*
Histamine	0.131±0.020	0.131±0.015	NS
Inosine	0.280±0.032	0.230±0.030	*
Isoleucine	0.345±0.064	0.339±0.048	NS
Lactate	10.956±0.820	9.267±1.091	*
Leucine	1.501±0.267	1.591±0.254	NS
Malate	0.287±0.038	0.326±0.071	NS
Methionine	0.278±0.035	0.658±0.244	**
Methylguanidine	0.031±0.005	0.025±0.007	NS
Niacinamide	0.173±0.020	0.147±0.017	*
N-Methylhydantoin	0.179±0.025	0.160±0.013	NS
Phenylalanine	0.174±0.029	0.173±0.027	NS
Lysine	0.826±0.123	0.866±0.113	NS
sarcosine	0.114±0.023	0.110±0.018	NS
Succinate	0.741±0.151	0.621±0.223	NS
Trimethylamine	0.102±0.023	0.085±0.009	NS
Tyrosine	0.180±0.034	0.163±0.023	NS
Valine	0.558±0.101	0.599±0.095	NS
β-Alanine	0.431±0.051	0.471±0.054	NS
Acetone	0.047±0.007	0.061±0.013	*
N,N-Dimethylglycine	0.029±0.006	0.023±0.003	*
Ornithine	0.483±0.066	0.484±0.041	NS
Glycine	1.379±0.167	1.231±0.120	NS
Ascorbate	0.213±0.029	0.147±0.030	**
Mannose	0.188±0.060	0.175±0.049	NS

Glycerophosphocholine	2.469±1.082	1.550±0.484	NS
uridine	0.300±0.065	0.175±0.028	**
uracil	0.053±0.013	0.057±0.008	NS
Adenosine 5'-monophosphate	0.099±0.036	0.065±0.038	NS
Dihydrouracil	0.483±0.053	1.022±0.331	**

Note: NS, $p > 0.05$; *, $p < 0.05$; **, $p < 0.01$; ***, $p < 0.001$ for hypoxia liver *vs.* normoxic liver as determined the Student's t test. Red and blue colors denote that the difference is positive (i.e. A was increased compared to B) and negative, respectively.

Supplementary Table S2. Comparison of metabolite levels between two groups of mice based on relative integrals calculated from the 1D ^{1}H NMR spectra of kidney.

Metabolites	Mean ± standard deviation		Hypoxia
	Normoxic kidney	Hypoxic kidney	*vs.* Normoxia
Threonine	0.458±0.502	0.431±0.095	NS
O-Phosphocholine	1.364±0.181	0.978±0.249	**
Pantothenate	0.080±0.006	0.119±0.042	NS
Isoleucine	0.249±0.039	0.317±0.061	*
Leucine	1.108±0.193	1.398±0.282	*
Valine	0.433±0.072	0.576±0.119	*
3-Hydroxyisobutyrate	0.028±0.004	0.042±0.008	**
Ethanol	0.176±0.044	0.164±0.030	NS
3-Hydroxybutyrate	0.112±0.016	0.147±0.023	**
Lactate	6.205±0.948	6.822±1.751	NS
2-Phenylpropionate	0.154±0.019	0.172±0.028	NS
Alanine	1.211±0.166	1.482±0.301	NS
Acetate	0.390±0.108	0.367±0.082	NS
Glutamate	2.938±0.561	2.297±0.496	*
Methionine	0.310±0.032	0.371±0.073	NS
Succinate	0.570±0.112	0.543±0.157	NS
5,6-Dihydrouracil	0.378±0.037	0.452±0.080	NS
Aspartate	0.217±0.022	0.214±0.041	NS
Asparagine	0.196±0.030	0.216±0.044	NS
Trimethylamine	0.068±0.020	0.054±0.012	NS
Dimethylamine	0.049±0.006	0.049±0.001	NS
N,N-Dimethylglycine	0.031±0.005	0.045±0.010	**
N-Methylhydantoin	0.020±0.003	0.022±0.003	NS
Creatine phosphate	0.759±0.155	1.028±0.218	*
Creatinine	0.164±0.020	0.196±0.035	NS
N,N-Dimethylformamide	0.111±0.012	0.124±0.021	NS
Malonate	0.199±0.027	0.221±0.061	NS
Ethanolamine	0.663±0.083	0.681±0.163	NS
Choline	7.272±0.706	5.958±1.616	NS
Cystine	0.406±0.063	0.338±0.073	NS
Betaine	3.096±0.547	3.995±0.789	*
sn-Glycero-3-phosphocholine	2.553±0.473	2.189±0.468	NS
myo-Inositol	2.594±0.355	2.481±0.488	NS
Taurine	4.160±0.561	3.872±0.951	NS
Methanol	1.642±0.844	0.903±0.488	NS
Glycine	2.569±0.422	2.176±0.460	NS
Glycerol	1.263±0.165	1.255±0.353	NS
Glucose	0.361±0.116	0.422±0.172	NS
Inosine	0.535±0.046	0.537±0.108	NS

Mannose	0.063±0.018	0.066±0.017	NS
Glucuronate	0.029±0.005	0.039±0.021	NS
Allantoin	0.043±0.006	0.061±0.015	*
Uracil	0.089±0.012	0.095±0.021	NS
Uridine	0.237±0.022	0.251±0.058	NS
Nicotinamide adenine dinucleotide	0.011±0.001	0.012±0.002	NS
Fumarate	0.031±0.005	0.030±0.005	NS
Tyrosine	0.174±0.029	0.214±0.043	NS
Histamine	0.064±0.010	0.075±0.018	NS
Anserine	0.011±0.004	0.008±0.002	NS
Phenylalanine	0.392±0.070	0.482±0.112	NS
Imidazole	0.032±0.003	0.036±0.009	NS
Niacinamide	0.137±0.015	0.143±0.030	NS
Tryptophan	0.019±0.004	0.024±0.006	NS
Xanthine	0.109±0.013	0.111±0.030	NS
4-Pyridoxate	0.072±0.012	0.078±0.042	NS
3-Methylxanthine	0.015±0.004	0.018±0.003	NS
Adenine	0.662±0.123	0.696±0.133	NS
Formate	0.008±0.002	0.008±0.001	NS
Inosinic acid	0.011±0.002	0.011±0.003	NS

Note: NS, $p > 0.05$; *, $p < 0.05$; **, $p < 0.01$; ***, $p < 0.001$ for hypoxic kidney *vs.* normoxic kidney as determined by the Student's t test. Red and blue colors denote that the difference is positive (i.e. A was increased compared to B) and negative, respectively.

Table S3. Comparison of metabolite levels between two groups of mice based on relative integrals calculated from the 1D ^{1}H NMR spectra of brain.

Metabolites	Mean ± standard deviation		Hypoxia
	Normoxic brain	Hypoxic brain	*vs*. Normoxia
Isoleucine	0.071±0.005	0.082±0.009	*
Leucine	0.255±0.019	0.298±0.028	**
Valine	0.127±0.005	0.134±0.016	NS
3-Hydroxyisobutyrate	0.039±0.005	0.039±0.006	NS
Ethanol	0.541±0.228	0.456±0.216	NS
3-Hydroxybutyrate	0.119±0.029	0.148±0.032	NS
Lactate	9.252±1.322	7.779±1.010	NS
Alanine	0.650±0.086	0.655±0.074	NS
4-Aminobutyrate	2.324±0.213	2.678±0.297	*
Acetate	0.667±0.101	0.769±0.116	NS
N-Acetylaspartate	2.571±0.177	2.584±0.190	NS
Glutamate	2.790±0.131	3.173±0.199	**
Glutamine	1.686±0.165	1.545±0.125	NS
Succinate	0.171±0.042	0.232±0.047	*
Aspartate	0.648±0.259	0.739±0.047	**
Creatine phosphate	4.693±0.184	4.765±0.441	NS
Taurine	3.292±0.350	3.122±0.325	NS
O-Phosphocholine	0.585±0.081	1.111±0.140	***
sn-Glycero-3-phosphocholine	1.123±0.076	1.215±0.127	NS
Myo-Inositol	2.072±0.072	2.240±0.132	*
Caffeine	0.182±0.262	0.160±0.037	NS
Glycine	0.798±0.060	0.683±0.071	*
Glycerol	0.824±0.059	0.746±0.064	*
Ascorbate	0.277±0.019	0.277±0.029	NS
Glucose	0.008±0.006	0.005±0.003	NS
Uracil	0.021±0.003	0.024±0.003	NS
Uridine	0.052±0.001	0.050±0.003	NS
Guanosine triphosphate	0.020±0.002	0.023±0.002	*
Inosine	0.304±0.016	0.312±0.017	NS
Fumarate	0.059±0.009	0.061±0.004	NS
Tyrosine	0.063±0.005	0.064±0.007	NS
Histamine	0.015±0.002	0.019±0.002	*
Adenosine	0.015±0.002	0.018±0.005	NS
Inosinic acid	0.023±0.005	0.028±0.008	NS
Formate	0.004±0.001	0.006±0.001	***
Adenine	0.120±0.013	0.1189±0.026	NS

Adenosine monophosphate	0.023±0.005	0.028±0.008	NS
Niacinamide	0.056±0.002	0.062±0.004	**
Nicotinamide adenine dinucleotide	0.003±0.002	0.006±0.001	**

Note: NS, $p > 0.05$; *, $p < 0.05$; **, $p < 0.01$; ***, $p < 0.001$ for hypoxic brain *vs*. normoxic brain as determined by the Student's t test. Red and blue colors denote that the difference is positive (i.e. A was increased compared to B) and negative, respectively.
